# Effects of iron intake on neurobehavioural outcomes in African children: a systematic review and meta-analysis of randomised controlled trials

**DOI:** 10.12688/wellcomeopenres.16931.2

**Published:** 2021-11-24

**Authors:** Agnes M. Mutua, Kelvinson Mwangi, Amina Abubakar, Sarah H. Atkinson

**Affiliations:** 1 Kenya Medical Research Institute (KEMRI), Centre for Geographic Medicine Research-Coast, KEMRI Wellcome Trust Research Programme, Kilifi, 230-80108, Kenya; 2 Institute for Human Development, Aga Khan University, Nairobi, 30270-00100, Kenya; 3 Department of Psychiatry, University of Oxford, Oxford, OX3 7JX, UK; 4 Department of Public Health, School of Human and Health Sciences, Pwani University, Kilifi, 195-80108, Kenya; 5 Centre for Tropical Medicine and Global Health, Nuffield Department of Medicine, University of Oxford, Oxford, OX3 7FZ, UK; 6 Department of Paediatrics, University of Oxford, Oxford, OX3 9DU, UK

**Keywords:** Iron deficiency, iron deficiency anaemia, African children, cognitive, motor, language, behaviour, development.

## Abstract

**Background:** Iron deficiency and developmental delay are common in African children. While experimental studies indicate an important role of iron in brain development, effects of iron on child development remain unclear. We aimed to evaluate the effects of iron supplementation or fortification on neurobehavioural outcomes in African children and further summarise these effects in children living in non-African countries for comparison.

**Methods:** We searched PubMed, EMBASE, PsycINFO, Scopus and Cochrane Library for studies published up to 22 ^nd^ October 2021. We included randomised controlled trials (RCTs) evaluating effects of iron supplementation or fortification on neurobehavioural outcomes in children. Due to heterogeneity in study methods, we analysed all studies qualitatively and in secondary analyses only seven RCTs with 11 arms were meta-analysed.

**Results:** We identified 2231 studies and included 35 studies (n=9988) in the systematic review. Only five studies (n=1294) included African children while 30 (n=8694) included children living in non-African countries. Of the five African studies, two (n=647) reported beneficial effects of iron supplementation on neurobehavioural outcomes in anaemic children, while three (n=647) found no beneficial effects. Of 30 studies in children living in non-African countries, 10 (n=3105) reported beneficial effects of iron supplementation or fortification on neurobehavioural outcomes, seven (n=786) reported beneficial effects only in children who had iron deficiency, iron deficiency anaemia or anaemia while 13 (n=4803) reported no beneficial effects.

**Conclusions:** There are few studies in African children despite the high burden of iron deficiency and developmental delay in this population. Evidence on the effects of iron supplementation on neurobehavioural outcomes remains unclear and there is need for further well-powered studies evaluating these effects in African populations.

**PROSPERO registration:** CRD42018091278 (20/03/2018)

## Introduction

Iron deficiency is the most common micronutrient deficiency affecting about two billion individuals globally and accounting for over 40% of all cases of anaemia ^1– 3^. Children living in Africa disproportionately bear the highest burden of anaemia and iron deficiency. Approximately 43% of pre-school children are anaemic globally and over 60% of these children live in Africa ^1, 4^, while it is estimated that 52% of African children are iron deficient ^5^.

Brain development begins at conception and continues into early adulthood ^6^. During this period and particularly in the first five years of life, children living in Africa are vulnerable to impaired neurobehavioural development as a result of exposure to different risk factors including poverty, malnutrition and infectious diseases ^7, 8^. About a third of pre-school children in low and middle-income countries (LMICs) are unlikely to reach their cognitive and/or socioemotional milestones and 44% of these children live in sub-Saharan Africa ^9^. Long-term consequences of impaired child development include poor educational performance, low incomes and poor family planning, contributing to the cycle of poverty in LMICs ^8^.

Iron deficiency and anaemia are important risk factors for impaired brain development in childhood ^10, 11^. However, epidemiological studies provide inconclusive evidence for the effects of iron supplementation or fortification on neurobehavioural outcomes despite compelling evidence from animal and *in vitro* studies. These studies indicate that iron plays an important role in neurotransmission, DNA synthesis and myelinogenesis ^12– 14^. Iron is also important for the synthesis of tryptophan hydroxylase and tyrosine hydroxylase, enzymes that are involved in the synthesis of serotonin, dopamine and norepinephrine, which are important for neurobehavioural processes in the brain ^14, 15^. Iron deficiency is associated with long-term behavioural abnormalities and impaired dopaminergic-dependent synaptic plasticity in the hippocampus, which may result in learning and memory deficits ^16, 17^.

Despite the high prevalence of both iron deficiency and anaemia, there are few studies investigating the effects of giving iron on neurobehavioural outcomes in African children. This may be due to many factors including concern that iron supplementation may increase the risk of malaria and other infections ^18– 20^. In this systematic review and meta-analysis, our objective was to evaluate the effects of iron supplementation or fortification on neurobehavioural outcomes in children living in Africa. For comparison, we further summarised evidence from randomised controlled trials (RCTs) on the effects of iron supplementation on neurobehavioral outcomes in non-African countries.

## Methods

### Reporting guidelines

Our systematic review and meta-analyses were guided by the Preferred Reporting Items for Systematic Review and Meta-Analysis (PRISMA) guidelines ^21^ and the protocol was registered on the PROSPERO database on 20 ^th^ March 2018 (registration number CRD42018091278).

### Search strategy and eligibility criteria

We searched PubMed, EMBASE, PsycINFO, Scopus and Cochrane Library for studies published up to 22 ^nd^ October 2021. Additionally, we scanned reference lists of identified studies and previous systematic reviews. We conducted searches for RCTs using a search strategy combining Medical Subject Heading terms for [iron] AND [neurobehavioural outcomes] AND [children] AND [RCT ‘publication type’]. We modified the search strategy as appropriate for each of the specific databases (Extended data, file 1 ^21^). The search was not restricted by language or publication date.

We included studies that met the following criteria: (i) included participants aged below 18 years; (ii) RCTs of iron supplementation or fortification in children or pregnant mothers; (iii) assessed neurobehavioural outcomes in children including cognitive or motor development, intelligence quotient, attention, behaviour, educational achievement or language development. We excluded studies assessing neurobehavioural outcomes in adult participants and RCTs involving iron supplementation/fortification alongside other micronutrients or macronutrients that did not separately evaluate the effects of iron. We also excluded observational studies, reviews, case studies, abstracts, comments and study protocols.

### Study selection, data extraction and quality appraisal

Two authors (AMM and KM) independently screened titles and abstracts of all identified studies against the inclusion criteria and then screened identified full texts to determine eligibility for inclusion. Disagreements between reviewers on study methodologies were resolved through discussion following the pre-determined inclusion and exclusion criteria. We extracted the following variables: study author(s) and year of publication, country, sample size, baseline iron status, age at iron supplementation and neurobehavioural assessment, neurobehavioural domain assessed and the tools used, definition of iron status and findings of the study.

We used the revised Cochrane risk-of-bias tool for randomised trials (RoB 2) to assess for risk of bias for the individually-randomised, parallel-group RCTs included in the review ^22^. RoB 2 assesses five domains of bias including bias from the randomization process, deviations from intended interventions, missing outcome data, and bias in measurement of the outcome and selection of the reported result. To assess the risk of bias in two cluster RCTs, we used the revised Cochrane risk of bias tool for randomised trials with additional considerations for cluster-randomised trials ^23^.

### Synthesis of included studies

The large degree of diversity in the study variables necessitated narrative synthesis of the study findings for all included studies. We grouped and discussed the studies based on the neurobehavioural domain assessed and summarised study characteristics and findings ( Table 1 and Extended data, file 2 ^21^). Under each neurobehavioural domain, we first summarised studies in African countries followed by studies in non-African countries. We compared findings of studies from African and non-African countries and also studies that evaluated the effect of iron-fortified foods compared to non-fortified foods. We further compared study findings based on age (studies in infants versus older children) and baseline iron status (normal iron status versus iron deficiency, iron deficiency anaemia or anaemia).

**Table 1.** Summary of studies assessing the effect of iron supplementation or fortification on neurobehavioural outcomes in African children: characteristics and findings.

Author, year (country)	Sample	Baseline iron status	Age at iron supplementation	Age at neuroassessment	Domain (assessment tool)	Intervention (duration)	Definition of iron status	Results
Ssemata, 2020 (Uganda) ^24^	N=145 with cerebral malaria (75 received iron concurrently and 70 received iron 28 days after antimalarial treatment)	All children had ID	18 to 58.8 months	At 3 time-points: 18 months to 4.9 years, 24 to 64.8 months and 30 to 70.8 months	Cognitive Executive function Sustained attention Associative memory Socioemotional behaviour (MSEL, ECVT, COAT, CBCL, BRIEF-P, BRS)	Ferrous sulphate 2mg/kg/ day either concurrently with antimalarial treatment or 28 days after receiving antimalarial treatment (3 months)	ID: ZnPP ≥ 80 µmol/mol heme	No difference in neurobehavioural scores between children who received iron supplementation concurrently or 28 days after antimalarial treatment.
Bouhouch, 2016 (Morocco) ^25^	n=455 lead exposed children (110 received iron, 116 received iron+ EDTA, 112 received EDTA, and 117 received placebo)	Anaemia: 21% ID: 32%, 7% or 34% as defined by SF, TfR, or ZnPP	3 to 14 years	At 2 time-points: at baseline (3 to 14 years) and after supplementation.	Cognitive Memory (KABC- II, HVLT)	2-3 biscuits (depending on body weight) containing 8 mg ferrous sulphate, 8 mg ferrous sulphate + 41 mg EDTA, 41 mg EDTA, or placebo (28 weeks)	ID: SF <12 mg/L for children <5 years, SF <15 mg/L for children ≥5 years, or TfR >8.3 mg/L with (CRP ≤5 mg/L, α1-acid glycoprotein ≤51 g/L) Anaemia: Hb<11.0 g/dL for children <5 years, Hb<11.5 g/dL for children 5–11 years, Hb <12.0 g/dL for children 12 years	No difference in cognitive or memory scores between children who received iron supplementation or placebo.
Baumgartner, 2012 (South Africa) ^26^	n=288 (70 received iron+ placebo, 72 placebo + DHA/EPA, 73 iron+ DHA/EPA, and 73 placebo + placebo)	Anaemia: 20.6-21.1% ID: 6.2% to 16%	6 to 11 years	At 2 time-points: at baseline (6 to 11 years) and after supplementation.	Cognitive Memory (HVLT KABC)	50 mg iron sulphate +DHA/ EPA (420/80 mg), 50 mg iron sulphate+ placebo, placebo+ DHA/ EPA, or placebo + placebo (8.5 months)	ID: SF<15 µg/L excluding children with CRP >5 mg/L or ZnPP >70 µmol/mol or TfR >8.3 mg/L Anaemia: Hb <11.5 g/dL IDA: anaemia + SF<15 µg/L	Anaemic children who received iron supplementation+ placebo had higher cognitive and memory scores compared to children who received placebo + placebo No difference in cognitive scores in children who received iron+ DHA/EPA compared to children who received placebo + placebo
Stoltzfus, 2001 (Zanzibar) ^27^	n=359 (183 received iron and 176 placebo)	Anaemia: 97% Severe anaemia: 18%	6 to 59 months	At 2 timepoints: 6 to 59 months and 18 to 71 months	Language Motor (Parents reported motor and language milestones)	20 mg ferrous sulphate or placebo (12 months)	Anaemia: Hb<110g/l Severe anaemia: Hb<7 g/dL ID: SF<12 mg/L	Children who received iron supplementation had improved language scores compared to children who received placebo and children with baseline Hb<9 g/dL who received iron supplementation had improved motor scores compared to children who received placebo.
Boivin, 1993 (Zaire) ^28^	N=47 (17 children received both anthelminthics and iron, 7 only iron, 8 only anthelminthics, and 15 did not receive either intervention)	Not indicated	23 boys (mean age=7.7, SD=0.8 years) and 24 girls (mean age=8.0, SD =1.8 years)	At baseline (mean age for boys=7.7, SD=0.8 years) and mean age for girls=8.0, SD =1.8 years) and 4 weeks after the 1st assessment	Cognitive (KABC)	20 mg iron (4 weeks)	Anaemia: Hb<12 g/dL	No difference in cognitive scores between children who received only iron supplementation or placebo.

BRIEF-P, Behavior Rating Inventory of Executive Functioning, Preschool edition; BRS, Behavior Rating Scales; CBLC, Child Behaviour checklist; CRP, C-reactive protein; COAT, Color Object Association Test; DHA/EPA, docosahexaenoic acid and eicosapentaenoic acid; ECVT, Early Childhood Vigilance Test; EDTA, ethylenediaminetetraacetic acid; Hb, haemoglobin; HVLT, Hopkins Verbal Learning Test; ID, iron deficiency; KABC, Kaufman Assessment Battery for Children; SD, standard deviation; SF, serum ferritin; TfR, transferrin receptor; ZnPP, zinc protoporphyrin.

### Secondary meta-analysis of seven studies in non-African countries.

Due to the substantial variation in study methods, we did a secondary meta-analysis in a limited number of RCTs that all used the Bayley Scales of Infant Development (BSID) to assess cognitive and motor development in children living in non-African countries. None of the studies in African children used the BSID. In the meta-analysed studies, mean cognitive and motor development scores and standard deviations were reported. For each of the two domains, we generated forest plots to show the mean differences (MDs) and the weight of each study and the pooled effect size with their corresponding 95% confidence intervals (CIs). Heterogeneity between the studies was assessed using the I ^2^ statistic. We applied random-effects meta-analysis since the I ^2^ values were > 40%. All analyses were conducted using STATA version 15.1 (StataCorp, College Station, TX 77845, USA).

## Results

### Study selection

**Figure 1.**
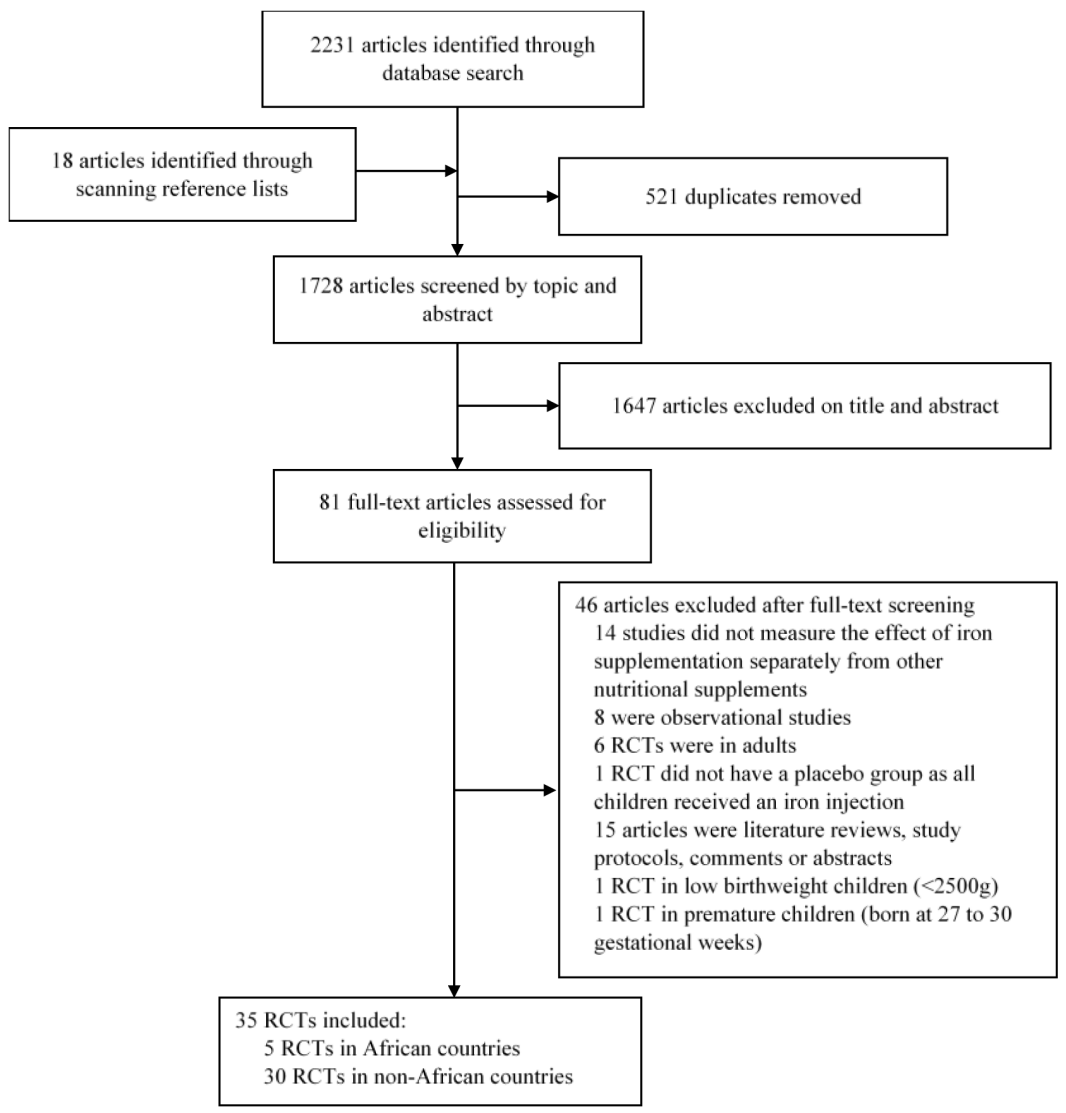
PRISMA flow chart showing the selection process for studies included in the review and meta-analysis.

We identified a total of 2231 papers from the database searches and an additional 18 papers from screening references of eligible papers ( Figure 1). We removed 521 duplicates and after screening titles and abstracts, excluded 1647 papers that were not relevant to our review. We further excluded 14 RCTs because participants were randomised to multiple micronutrient powders or other nutritional supplements together with iron and the effect of iron supplementation was not evaluated separately from the other supplements. We excluded eight observational studies, six RCTs in adults and 15 papers that were literature reviews, study protocols, comments or abstracts. We excluded one study that did not have a placebo group as all participants received a single iron-dextran intramuscular injection. We further excluded one RCT in low birthweight children (<2500g) and another in premature children (born at 27 to 30 gestational weeks).

### Study characteristics and outcomes

**Figure 2.**
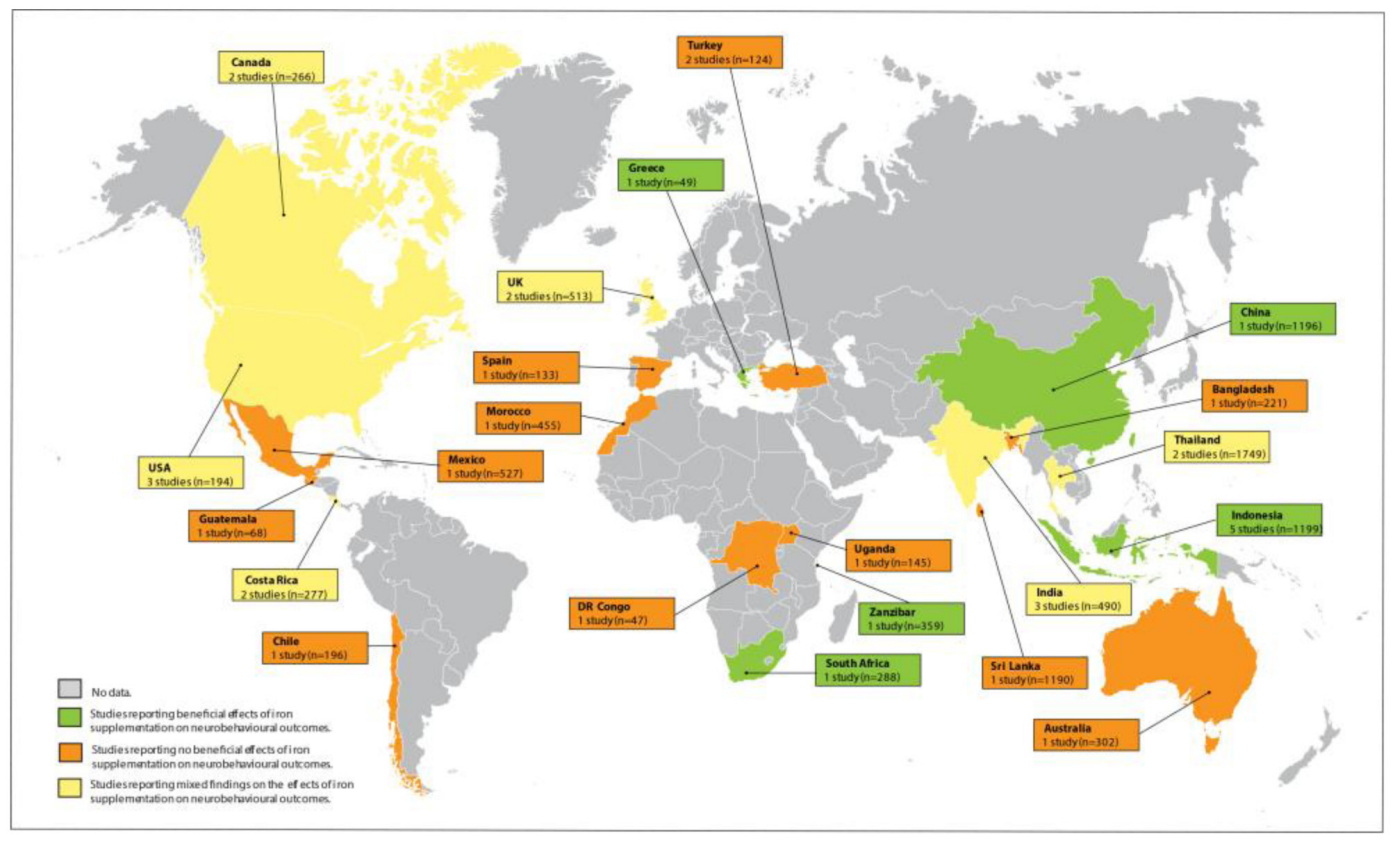
Studies of the effect of iron supplementation or fortification on neurobehavioural outcomes in children. Five studies (n=1294) were in Africa, 15 (n=6169) in Asia, four (n=695) in Europe, nine (n=1332) in North America, one (n=196) in South America and one (n=302) in Australia.

We included a total of 35 RCTs published between 1978 and 2021 ( Figure 2). In total, five RCTs were in African countries while 30 RCTs were in non-African countries. Among the 35 RCTs were those that studied the effect of: iron supplementation compared to placebo or no treatment (n=25), iron-fortified foods compared to non-fortified foods (n=5), formula milk fortified with high compared to low dosages of iron (n=1), immediate iron supplementation given concurrently or 28 days after antimalarial treatment on development in children with severe malaria (n=1), varying and consistent doses of iron supplementation compared to placebo (n=1), and maternal iron supplementation on neurobehavioural outcomes in children after birth (n=2). Out of the 35 studies, two were in lead-exposed children at baseline. Overall, 11 studies were carried out in high-income countries and 24 in low and middle-income countries. The sample sizes ranged from 16 to 1358 and the RCTs provided varying forms of iron supplementation in varying dosages over periods ranging from 1 day to 15 months. The studies evaluated various neurobehavioural outcomes including cognitive, motor, language and behavioural development and educational achievement using a wide range of neuroassessment tools, the most common being the Bayley Scales of Infant Development. Iron status and anaemia were defined differently in the studies using varying iron biomarkers and haemoglobin (Hb) cut-offs. The characteristics of the included studies are shown in Table 1 and Extended data, file 2 ^21^. Of the 35 RCTs, 19 showed a low risk of bias, one showed a high risk of bias, while 15 studies were judged to raise some concerns (Extended data, files 3 and 4 ^21^). Some common limitations included a lack of description of the randomisation process and missing outcome data.

### Cognitive development

A total of 28 RCTs (n=7503) investigated the effect of iron supplementation (n=23) or fortification (n=5) on cognitive development. Out of these studies, four (n=935) were in African countries including two among children living in sub-Saharan Africa (n=433). One RCT of 288 South African children aged six to 11 years reported improved cognitive and memory scores among anaemic children Hb<11.5 g/dL) who received iron supplementation for 8.5 months compared to children who received placebo ^26^. However, two RCTs, one in 455 lead-exposed Moroccan children aged three to 14 years and another in 47 Zairean school children aged seven to nine years, reported that iron supplementation or fortification for four to 28 weeks was not beneficial for cognitive development ^25, 28^. One RCT (n=145) without a placebo arm reported no improvement in cognitive development in Ugandan pre-school children with severe malaria who received iron supplementation concurrently or 28 days after antimalarial treatment ^24^.

In comparison, a total of 24 RCTs (n=6568) investigated the effect of iron supplementation or fortification on cognitive development in children living in non-African countries. Out of these 24 studies, three (n=604) reported beneficial effects on cognitive development, six (n=678) reported beneficial effects only in children with iron deficiency anaemia (IDA) at baseline, while 15 found no beneficial effects. Of the three studies reporting beneficial effects, one RCT of 73 adolescent girls in the USA reported improved verbal learning and memory after iron supplementation for eight weeks compared to placebo ^29^, another RCT of 391 Thai children reported improved intelligence quotient (IQ) scores in children who received once-a-week iron supplementation compared to those who received daily iron supplementation or placebo for 16 weeks ^30^, while one RCT of 140 Indian adolescents reported improved cognition in children who received iron-biofortified pearl millet compared to those who received conventional pearl millet ^31^. Six studies reported improved cognitive development among children with IDA or anaemia at baseline. Of these six studies, four RCTs, two in Indonesia (n=295), one in Costa Rica (n=191) and one in Greece (n=49), reported improved cognitive development after iron supplementation for two to four months in pre-school children ^32– 35^. Another RCT in 24 pre-school American children with IDA reported improved cognitive development among those who received a single iron-dextran complex injection compared to a single sterile saline injection ^36^. One RCT (n=119) in Indonesia reported improved IQ among school children with IDA who received iron supplementation for three months compared to those who received placebo ^37^.

In total, 15 (n=5286) out of 24 studies found no beneficial effects of iron supplementation or fortification on cognitive development. Among pre-school children, seven RCTs, two in Turkey (n=124), one in Indonesia (n=655), one in Bangladesh (n=221), one in Chile (n=196), one in Costa Rica (n=86) and one in Guatemala (n=68) reported no beneficial effects of iron supplementation for one week to six months on cognitive development ^38– 44^. Three RCTs in Canada (n=225), the UK (n=428) and Spain (n=133) reported no beneficial effects of iron-fortified formula on cognitive development in children aged six to nine months compared to unfortified formula milk, low-iron fortified formula or cow’s milk ^45– 47^. Among school children three RCTs, one in Thailand (n=1358), one in Sri-Lanka (n=1190) and one in Indonesia (n=130), reported that iron supplementation for three to six months was not beneficial for cognition in children aged eight to 11.6 years ^33, 48, 49^. One RCT of 170 Indian adolescents found no beneficial effect of daily iron-fortified wheat-based meals for seven months on cognition compared to unfortified wheat-based meals ^50^. Another RCT in 302 Australian mother-child pairs reported no beneficial effects of iron supplementation during pregnancy on IQ at four years ^51^. Secondary meta-analysis of seven studies (n=775) provided limited evidence of beneficial effects of iron supplementation on cognitive development in pre-school children (MD=1.73, 95% CI, -1.05, 4.52) (Extended data, file 5 ^21^).

### Motor development

A total of 19 RCTs (n=4438) investigated the effect of iron supplementation (n=16) or fortification (n=3) on motor outcomes. Out of these studies, only one was in African children. One RCT of 359 children in Zanzibar reported improved motor and language scores in children with Hb<9 g/dL at baseline who received iron supplementation for 12 months compared to children who received placebo ^27^.

A total of 18 RCTs (n=4079) investigated the effect of iron supplementation or fortification on motor development in children living in non-African countries. Out of these 18 studies, seven (n=2479) reported beneficial effects on motor development, two (n=310) reported beneficial effects only in children with IDA at baseline, while nine (n=1290) found no beneficial effects. Of the seven studies reporting beneficial effects, one RCT of 1196 Chinese children aged six weeks whose mothers also received iron supplementation during pregnancy reported that iron supplementation for 7.5 months, with or without iron supplementation in pregnancy, improved gross motor development compared to placebo ^52^. Four RCTs, one in Indonesia (n=655), one in India (n=180), one in the USA (n=97) and another in Canada (n=41) reported beneficial effects of iron supplementation for two to six months on motor development compared to placebo ^38, 53– 55^. Two RCTs, one in the UK (n=85) and another in Canada (n=225) reported improved motor development in children aged six to eight months who received iron-fortified formula milk for 10 to 15 months compared to children who received regular formula or cow’s milk ^47, 56^. Two studies reported improved motor development after iron supplementation among children with IDA at baseline. Two RCTs, one in Costa Rica (n=191) and one in Indonesia (n=119), reported that pre-school children with IDA who received iron supplementation for three to four months had improved motor development compared to children who received placebo ^33, 35^.

Out of the 18 studies, nine found no beneficial effects of iron supplementation or fortification on motor development in pre-school children. Six RCTs, two in Turkey (n=124), one in Chile (n=196), one in Bangladesh (n=221), one in Costa Rica (n=96) and one in Guatemala (n=68) reported no beneficial effect of three to six months of iron supplementation compared to placebo ^39– 44^. Another small RCT in 24 pre-school American children with IDA reported no beneficial effects of a single iron-dextran complex injection on motor development ^36^. Two RCTs, in the UK (n=428) and Spain (n=133) reported no beneficial effects of iron-fortified formula milk on motor development compared to unfortified formula, cow’s milk or low-iron formula milk ^45, 46^. The seven studies (n=775) included in a secondary meta-analysis showed no beneficial effects of iron supplementation on motor development in pre-school children (MD=1.99, 95% CI, -0.97, 4.95) (Extended data, file 6 ^21^).

### Behavioural functioning

In total, eight RCTs (n=2295) investigated the effect of iron supplementation (n=7) or fortification (n=1) on behavioural functioning in children. Of these eight studies, only one (n=145) was in African children. One RCT of 145 Ugandan children aged 18 to 59 months with severe malaria reported no improvement in behavioural functioning in children who received iron supplementation concurrently or 28 days after antimalarial treatment ^24^. A total of seven RCTs (n=2150) investigated the effect of iron supplementation or fortification on behavioural functioning in children living in non-African countries. Of these seven studies, one (n=24) reported beneficial effects on behavioural functioning in children with IDA, while six (n=2126) reported no beneficial effects. One small RCT of 24 American pre-school children with IDA reported improved behavioural functioning among children who received a single iron-dextran complex injection ^36^. Of the six studies reporting no effect, three, one in Indonesia (n=655), one in Bangladesh (n=221) and one in Chile (n=196), reported no beneficial effects of iron supplementation for three to six months on behavioural functioning in children up to six months of age ^38, 39, 43^. One RCT of 527 lead-exposed Mexican children similarly reported no beneficial effect of iron supplementation for six months on behavioural functioning ^57^. Another RCT of 225 Canadian children aged six months found no beneficial effects of iron-fortified formula for 15 months on behaviour compared to regular formula ^47^, while one RCT in 302 Australian mother-child pairs reported no beneficial effects of iron supplementation during pregnancy on behaviour at four years ^51^.

### Educational achievement

Overall, five RCTs (n=3188) investigated the effect of iron supplementation on educational achievement in children living in non-African countries. There were no studies in African countries. One RCT of 130 Indonesian children aged 8 to 12 years reported improved educational achievement in anaemic children who received iron supplementation for three months compared to those who received placebo ^58^. Out of the five studies, four (n=3058) found no beneficial effects. Three RCTs, one in Sri Lanka (n=1190), one in Thailand (n=1358) and one in Indonesia (n=119) found no beneficial effects of iron supplementation for three to six months on educational achievement ^37, 48, 49^, while another RCT including 391 Thai school children reported no beneficial effect of once-a-week or daily iron supplementation for 16 weeks on educational achievement compared to placebo ^30^.

### Effect of iron supplementation based on baseline iron status

Out of 18 studies (n=3524), 11 (n=2769) evaluated the effect of iron supplementation in groups of children with ID, IDA or anaemia compared to children with normal iron status at baseline, five (n=698) included only children with ID, IDA or anaemia at baseline, while two (n=57) included only children with sufficient iron status at baseline. Of the 11 studies that compared groups, six (n=953) reported beneficial effects of iron supplementation on neurobehavioural outcomes in children with ID, IDA or anemia compared to those with normal iron status ^26, 32– 35, 58^, while five (n=1816) reported no beneficial effects ^40, 42– 44, 49^. Of the five studies that included only children with ID, IDA or anaemia at baseline, four (n=553) reported beneficial effects of iron supplementation on neurobehavioural outcomes ^27, 29, 36, 54^, while one (n=145) found no beneficial effects ^24^. Secondary sub-group meta-analysis based on baseline iron status in studies using the BSID indicated that iron supplementation was not beneficial for cognitive or motor development (Extended data, file 5 and 6 ^21^). However, these sub-group analyses were limited by the small sample sizes.

### Effect of iron supplementation or fortification in children during infancy versus older age

Overall, ten RCTs (n=3180) evaluated the effect of iron supplementation (n=6) or fortification (n=4) on neurobehavioural outcomes during infancy and of these studies, six (n=2382) reported beneficial effects of iron supplementation (n=4) or fortification (n=2) ^38, 43, 52, 53, 55, 56^, while four (n=798) found no beneficial effects of iron supplementation (n=2) or fortification (n=2) ^39, 41, 45, 46^. In children above one year of age, 24 RCTs (n=6506) evaluated the effects of iron supplementation (n=22) or fortification (n=2) on neurobehavioural outcomes and of these, 13 (n=2156) reported a beneficial effect of iron supplementation (n=12) or fortification (n=1) on neurobehavioural outcomes ^26, 27, 29– 37, 54, 58^ while 11 (n=4350) reported no beneficial effects of iron supplementation (n=10) or fortification (n=1) ^24, 25, 28, 40, 42– 44, 48– 50, 57^.

### Effect of duration of supplementation or fortification

In total, seven studies (n=534) investigated the effect of iron supplementation for less than three months and of these studies, five (n=419) reported beneficial effects of iron supplementation on neurobehavioural outcomes ^29, 32, 34, 54^, while two (n=115) reported no beneficial effects ^28, 44^. Out of 26 studies (n=8972) that evaluated the effect of iron supplementation (n=20) or fortification (n=6) for three months or more, 13 (n=3939) reported beneficial effects of iron supplementation (n=10) or fortification (n=3) on neurobehavioural outcomes ^26, 27, 30, 31, 33, 35, 37, 38, 47, 52, 53, 56, 58^ and 13 (n=5033) reported no beneficial effects of iron supplementation (n=10) or fortification (n=3) ^24, 25, 39– 43, 45, 46, 48– 50, 57^.

### Comparison of studies in African countries versus non-African countries

Five RCTs including a total of 1294 children evaluated the effect of iron supplementation or fortification on cognitive (n=4), motor (n=1), behavioural (n=1) or language (n=1) outcomes in African children. Only three out of the five studies were in sub-Saharan Africa and of the three one did not have a placebo group. Of the five studies, two (n=647) reported beneficial effects on cognitive, motor or language outcomes only in children with Hb<9 g/dL and <11.5 g/dL compared to children with higher Hb levels ^26, 27^, while three (n=647) reported no beneficial effects ^24, 25, 28^. Of the three studies that reported no beneficial effect of iron supplementation, one included only children with ID ^24^, one had a prevalence of 21% of anaemia ^25 ^while one did not report baseline iron status ^25^. Among children living in non-African countries, 30 RCT (n=8694) investigated the effect of iron supplementation or fortification on neurobehavioural outcomes. Compared to studies in African children, over half of the 30 studies reported beneficial effects on neurobehavioural outcomes, mostly in children with ID, IDA or anaemia ^29– 54, 56– 58^.

## Discussion

In this systematic review and meta-analysis, we found mixed evidence for the effects of iron supplementation or fortification on neurobehavioural outcomes in children. Few studies have investigated the effects of iron supplementation on neurobehavioural outcomes in African children despite the high burden of both iron deficiency and developmental delay in this population. Evidence from other regions on the effects of iron on neurobehavioural outcomes may not be generalisable to African children as these effects may be mediated by different risk factors such as malnutrition and a high burden of infectious diseases including malaria, HIV, tuberculosis and helminthic infections ^59^. Of five studies in African children only three included children living in sub-Saharan Africa, which has the highest prevalence of malaria ^60^, and one of these three studies had no placebo arm ^24^. Additionally, only six observational studies have evaluated the associations between iron status and neurobehavioural outcomes in African children and their findings are inconsistent ^61– 66^. While the World Health Organization recommends iron supplementation together with effective malaria treatment and prevention in children living in malaria-endemic areas, evidence on the optimal time for iron supplementation and its effects on neurobehavioural outcomes in African children is limited ^67^.

Little is known about the effects of iron supplementation or fortification on cognitive or language development and educational achievement in African children. Out of 5 studies we found mixed evidence for beneficial effects; two studies reported improved cognitive or language development in children with anaemia (Hb <9 g/dL and Hb <11.5 g/dL) compared to those with higher Hb levels ^22, 27^, while three studies reported lack of beneficial effects, one of these studies included only iron deficient children and two did not evaluate the effects of iron based on baseline iron status ^24, 25, 28^. No studies in Africa have investigated the effects of giving iron on educational achievement. Evidence from observational studies in African children is also limited with only three studies investigating associations between baseline iron status or anaemia and cognitive or language development. These three observational studies in Ethiopia, Egypt and Benin reported no association between child or maternal iron status and cognitive or language development in young children ^62, 64, 66^. Lack of associations in these studies may be explained by the sufficient iron status of the participants. Similarly, evidence for the effects of iron intake on cognitive development or educational achievement in children living in non-African countries was limited with only nine of 24 RCTs reporting beneficial effects, with six out of these nine in children with IDA. It is possible that some of the tools used to assess cognitive development detect broad aspects of cognition and may have limited sensitivity to smaller changes resulting from nutritional effects in specific elements of cognition such as attention and information processing speed ^68^. Effects of iron on aspects of cognition including concentration, memory, attention and IQ may mediate the reported improved educational achievement following iron supplementation in one study while the lack of beneficial effects observed in four of the 24 studies may be explained by the low prevalence of iron deficiency or anaemia at baseline ^48, 49^. In contrast to epidemiological studies, evidence from animal studies consistently suggests that iron may impact cognitive and language development through its roles in myelinisation, dopamine metabolism and the structure and function of the hippocampus, the centre for memory and learning processes ^16, 69, 70^.

Only a single study has evaluated the effects of iron supplementation on motor development in African children. This study in young Tanzanian children reported improved motor development in anaemic (Hb <9 g/dL) African children ^27^. Improved motor development following iron supplementation may be attributed to improved iron status. Iron deficiency is associated with low cellular oxygen-carrying capacity of blood in tissues causing low muscle energy production which may limit independent effort and balance delaying acquisition of motor skills in children ^71, 72^. Evidence from three observational studies in Zanzibar and Ghana reported that ID and/or IDA were associated with poor motor development in children ^61, 63, 65^. About half of the RCT studies in children living in non-African countries reported beneficial effects of iron supplementation on motor development. However, most of these studies did not explore the effects of iron supplementation based on iron status. The mixed findings may be explained by differences in study methods such as sample sizes and baseline iron status. Animal studies provide mechanistic evidence of how iron might influence motor development. Iron is important for myelination in the corticospinal and corticostriatal tracts, the main pathways for motor signals from the brain to the limbs ^73^. Additionally, iron plays an important role in dopamine function in the basal ganglia, an important area in the brain for motor function ^74^.

We found little evidence for the effects of iron supplementation on behavioural functioning. Only a single study has been conducted in African children and it reported no evidence of improved behavioural functioning in children who received iron supplementation concurrently or 28 days after antimalarial treatment. This study may be limited by the small sample size and lack of a placebo group. In children living in non-African countries, only one of five RCTs, in American children with IDA, reported beneficial effects. Children with IDA have been observed to be clumsy, inattentive, irritable and withdrawn, traits that are consistent with impaired behavioural functioning ^75^. The lack of beneficial effects of iron observed in some studies may be attributed to the small proportion of children with iron deficiency anaemia at baseline as mild iron deficiency may not result in depletion of iron in body tissues that would manifest in behavioural changes ^38, 43^. Evidence from animal studies indicate that iron may influence behavioural functioning through its role in dopaminergic neurotransmission which is key in behaviour activation and inhibition and reward seeking behaviour ^76 ^.

The mixed findings for the effects of iron supplementation on child development in our review may be attributed to differences in study methods, sensitivity of outcome measures used and populations. Based on baseline iron status, iron supplementation was mostly beneficial for development in children with iron deficiency or iron deficiency anaemia. Improvement of symptoms of iron deficiency or iron deficiency anemia, including lethargy and withdrawal, after iron supplementation may result in improved neurobehavioural outcomes in these children ^77^. Also, children with iron deficiency anaemia are likely to be fussy and clingy to their caregivers who may respond by holding them, which may delay the child’s independent exploration and interaction with their environment and consequently delay neurobehavioural development ^78^. We observed little difference when comparing findings between studies that gave iron supplementation during or after infancy. Evidence indicates that iron supplementation may be more beneficial to child development in early childhood when there is rapid brain development but beneficial effects of iron supplementation on neurobehavioural outcomes have also been reported in older children ^79– 81^. Only two studies evaluated the effects of maternal iron supplementation during pregnancy and they did not report beneficial effects on neurobehavioural outcomes in children after delivery ^51, 52^. One of the studies reported that maternal iron supplementation did not improve iron status in the newborn as indicated by cord blood ferritin at delivery, which may explain the lack of beneficial effects on child development ^82^. Further studies are necessary to evaluate the effects of maternal iron supplementation on neurobehavioural outcomes in children. We found little difference when comparing studies that gave iron supplementation for less than three months and studies that gave iron supplementation for three months or more. The World Health Organization recommends iron supplementation for three consecutive months in children living in areas with a high prevalence of anaemia for prevention of iron deficiency and anaemia, but it is unclear if this duration is adequate to improve neurobehavioural outcomes in children ^67^.

We identified five systematic reviews and meta-analyses of the effects of iron on neurobehavioural outcomes in children. Consistent with our review, one recent systematic review of 25 RCTs and 26 observational studies reported inconsistent findings for the effects of maternal or child iron supplementation or iron status on neurobehavioural outcomes in children ^83^. This systematic review only included studies with children below four years of age. Another systematic review and meta-analysis of 33 RCTs evaluating the effects of daily iron supplementation on child health reported no beneficial effects of iron supplementation on mental or psychomotor development in children aged four to 23 months ^84^. Of the 33 included studies, only six studies evaluated cognitive and psychomotor development in children. One systematic review and meta-analysis of 32 RCTs investigating the effects of daily supplementation on child health reported beneficial effects of iron supplementation on cognition and IQ among anaemic children and aspects of attention and concentration in children aged five to 12 years ^81^. Of the eligible 32 RCTs, only 12 assessed aspects of cognition in children and unlike our review, did not include studies that assessed other neurobehavioural domains. One Cochrane systematic review of eight RCTs assessing the effects of iron supplementation in children below three years of age who had iron deficiency reported no beneficial effects of iron supplementation on mental or psychomotor development ^85^. Another systematic review and meta-analysis of 14 RCTs evaluating the effects of oral iron supplementation in older school children and women reported beneficial effects of iron supplementation on attention, concentration and IQ but not memory, psychomotor function or school achievement ^79^.

## Strengths and limitations

Strengths of our review include a very comprehensive search strategy of five databases without restrictions in geographical location, language or date of publication. To our knowledge, our review is the first to summarise evidence on the effects of iron supplementation on neurobehavioural outcomes in African children in comparison to evidence in children living in non-African countries. Our review highlights that very few studies have been conducted in Africa, with only three conducted in sub-Saharan Africa. Additionally, our review included children up to the age of 18 years giving an overview of neurobehavioural outcomes across childhood. Limitations of our review were the inability to conduct a quantitative meta-analysis due to substantial heterogeneity in study populations and methods including the tools used to assess neurobehavioural outcomes and definitions for iron status. Our secondary meta-analyses are likely to be limited by small sample sizes and other unstandardized factors including age, dosage and duration of iron supplementation.

## Conclusions

We found conflicting evidence for the effects of iron supplementation or fortification on neurobehavioural outcomes in children and there were few studies in African children. Further, well-powered RCTs on the effects of iron supplementation on neurobehavioural outcomes in African children are needed considering the high burden of both iron deficiency and developmental delay in these populations. These studies further need to consider the impact of other risk factors such as infections and malnutrition on the relationship between iron and neurobehavioural outcomes in African children. Additionally, well-validated and standardised tools for assessing neurobehavioural outcomes across all age groups in childhood would help in comparison of findings in studies.

## Data availability

### Underlying data

All data underlying the results are available as part of the article and no additional source data are required.

### Extended data

Figshare: Effects of iron intake on neurobehavioural outcomes in African children: a systematic review and meta-analysis of randomised controlled trials-supplementary files. https://doi.org/10.6084/m9.figshare.14473077.v5 ^21^.

This project contains the following extended data:

- Extended datafile 1: Search terms.
- Extended datafile 2: Summary of studies assessing the effect of iron supplementation or fortification on neurobehavioural outcomes in children living in non-African countries: characteristics and findings.
- Extended datafile 3: Assessment of risk of bias in randomised parallel-group trials included in the review using the revised Cochrane risk-of-bias tool for randomised trials.
- Extended datafile 4: Assessment of risk of bias in cluster-randomised parallel-group trials included in the review using the revised Cochrane risk-of-bias tool for randomised trials with additional considerations for cluster-randomised trials.
- Extended datafile 5: Forest plot for the effects of iron supplementation on cognitive development: overall effect and subgroup analyses based on baseline iron status.
- Extended datafile 6: Forest plot for the effects of iron supplementation on motor development: overall effect and subgroup analyses based on baseline iron status.

### Reporting guidelines

Figshare: PRISMA checklist for Effects of iron intake on neurobehavioural outcomes in African children: a systematic review and meta-analysis of randomised controlled trials. https://doi.org/10.6084/m9.figshare.14473077.v5 ^21^.

Data are available under the terms of the Creative Commons Attribution 4.0 International license (CC-BY 4.0).
