# Supplementary material for: Effects of iron intake on neurobehavioural outcomes in African children: a systematic review and meta-analysis of randomised controlled trials
Source: Wellcome Open Res. 2021 Nov 24;6:181. Originally published 2021 Jul 13. [Version 2] doi: 10.12688/wellcomeopenres.16931.2 (PMC8777511; doi:10.12688/wellcomeopenres.16931.2)
Supplement: Peer review history

# Peer review: Reviewer response for version 2

**DOI:** 10.21956/wellcomeopenres.19259.r47217

**Published:** 2022-01-20

**Authors:** Michael Mireku

**Affiliations:** 1 School of Psychology, University of Lincoln, Lincoln, UK

I can confirm that the authors have sufficiently responded to my questions and made necessary changes that have improved the manuscript.

Are the rationale for, and objectives of, the Systematic Review clearly stated?

Yes

Is the statistical analysis and its interpretation appropriate?

Partly

Are sufficient details of the methods and analysis provided to allow replication by others?

Yes

Are the conclusions drawn adequately supported by the results presented in the review?

Yes

Reviewer Expertise:

NA

I confirm that I have read this submission and believe that I have an appropriate level of expertise to confirm that it is of an acceptable scientific standard.

# Peer review: Reviewer response for version 2

**DOI:** 10.21956/wellcomeopenres.19259.r47218

**Published:** 2021-11-25

**Authors:** Patricia Kitsao-Wekulo

**Affiliations:** 1 Maternal and Child Wellbeing Unit, African Population and Health Research Center, Nairobi, Kenya

I am satisfied with the changes made and have no further comments to make.

Are the rationale for, and objectives of, the Systematic Review clearly stated?

Yes

Is the statistical analysis and its interpretation appropriate?

I cannot comment. A qualified statistician is required.

Are sufficient details of the methods and analysis provided to allow replication by others?

Yes

Are the conclusions drawn adequately supported by the results presented in the review?

Yes

Reviewer Expertise:

Developmental psychology

I confirm that I have read this submission and believe that I have an appropriate level of expertise to confirm that it is of an acceptable scientific standard.

# Peer review: Reviewer response for version 1

**DOI:** 10.21956/wellcomeopenres.18683.r45469

**Published:** 2021-10-05

**Authors:** Michael Mireku

**Affiliations:** 1 School of Psychology, University of Lincoln, Lincoln, UK

Matua *et al*. have done excellent work on a very difficult topic. The authors attempted to investigate the effect of iron supplementation and fortification on neurobehavioural outcomes in children. The objective although clearly stated is very difficult to accurately investigate because of several reasons:

1. There are several domains of neurobehavioural outcomes assessed in most papers;
2. Different assessment scales are used across several studies and the scores generated from one scale are not easily interchangeable/transferable into another scale;
3. Some of these scales are adapted for use in specific countries and others are not;
4. Age-dependent dose of iron supplementation will vary for different settings. Trials that recruit only iron-deficient children may provide a relatively higher dose of iron relative to studies in a general, supposedly healthy population. Thus, the benefit of iron supplementation may be the same across groups because of the dose. the same can be said for fortification - how much of the fortified product is the child consuming? 

The authors have therefore done a very good job in identifying these potential issues and providing their analysis under these specific subheadings. 

 My only major concern is the validity of the meta-analysis in relation to the standardisation of the scales used to assess cognitive and motor functions. The authors failed to explain how they used the scores from the different scales in the meta-analysis.

 Further, findings from the meta-analysis are actually scanty for the obvious reason of heterogeneity across studies. This should be discussed further. 

**Minor comment:** Revise the first sentence under Methods "...metanalysis were" not "...metanalysis was"

Are the rationale for, and objectives of, the Systematic Review clearly stated?

Yes

Is the statistical analysis and its interpretation appropriate?

Partly

Are sufficient details of the methods and analysis provided to allow replication by others?

Yes

Are the conclusions drawn adequately supported by the results presented in the review?

Yes

Reviewer Expertise:

Perinatal and paediatric epidemiology; Sleep health

I confirm that I have read this submission and believe that I have an appropriate level of expertise to confirm that it is of an acceptable scientific standard, however I have significant reservations, as outlined above.

# Response

**Published:** 2021-11-16

**Authors:** Agnes Mutua

**Affiliations:** KEMRI-Wellcome Trust Research Programme, Kenya

We are grateful to the reviewer for his insightful comments and have addressed the below:

 Mutua *et al*. have done excellent work on a very difficult topic. The authors attempted to investigate the effect of iron supplementation and fortification on neurobehavioural outcomes in children. The objective although clearly stated is very difficult to accurately investigate because of several reasons:

1. There are several domains of neurobehavioural outcomes assessed in most papers;
2. Different assessment scales are used across several studies and the scores generated from one scale are not easily interchangeable/transferable into another scale;
3. Some of these scales are adapted for use in specific countries and others are not;
4. Age-dependent dose of iron supplementation will vary for different settings. Trials that recruit only iron-deficient children may provide a relatively higher dose of iron relative to studies in a general, supposedly healthy population. Thus, the benefit of iron supplementation may be the same across groups because of the dose. the same can be said for fortification - how much of the fortified product is the child consuming? 

The authors have therefore done a very good job in identifying these potential issues and providing their analysis under these specific subheadings. 

**My only major concern is the validity of the meta-analysis in relation to the standardisation of the scales used to assess cognitive and motor functions. The authors failed to explain how they used the scores from the different scales in the meta-analysis. Further, findings from the meta-analysis are actually scanty for the obvious reason of heterogeneity across studies. This should be discussed further.**

*Response: We thank the reviewer for this comment and agree that a main limitation of the review is the high level of heterogeneity in the studies which precluded a meta-analysis. We therefore analysed all studies in African and non-African countries qualitatively by summarizing the findings.*

*In secondary analyses we performed a meta-analysis of all studies (n=7) which used the same tool, the Bayley Scales of Infant Development, to assess cognitive and motor development. The Bayley Scales of Infant Development has standardized scales and therefore there was no need for any further standardization of the scores. We agree that these secondary analyses had a number of limitations. First, only a very limited number of studies used the Bayley Scales, which may introduce bias. Second, we were not able to meta-analyse African studies since this tool was not used and Bayley Scales are likely to be less applicable in this population. Third, although the meta-analysis included standardized assessment of neurodevelopmental outcomes many other factors were not standardized including dose and duration of iron supplementation.*

*We have now added a sub-heading to highlight the sub-section detailing the meta-analysis in the methods section as follows:*

*“Secondary meta-analysis of seven studies in non-African countries”*

*Due to the substantial variation in study methods, we did a secondary meta-analysis in a limited number of RCTs that all used the Bayley Scales of Infant Development (BSID) to assess cognitive and motor development in children living in non-African countries. None of the studies in African children used the BSID. In the meta-analysed studies, mean cognitive and motor development scores and standard deviations were reported. For each of the two domains, we generated forest plots to show the mean differences (MDs) and the weight of each study and the pooled effect size with their corresponding 95% confidence intervals (CIs). Heterogeneity between the studies was assessed using the I 2 statistic. We applied random-effects meta-analysis since the I 2 values were > 40%. All analyses were conducted using STATA version 15.1 (StataCorp, College Station, TX 77845, USA).”*

*We have summarized the results of the meta-analyis in the results section as follows:*

*‘Cognitive development’: “Secondary meta-analysis of seven studies (n=775), provided limited evidence of beneficial effects of iron supplementation on cognitive development in pre-school children (MD=1.73, 95% CI, -1.05, 4.52).”*

*‘Motor development’: “* *The seven studies(n=775) included in a secondary meta-analysis showed no beneficial effects of iron supplementation on motor development in pre-school children (MD=1.99, 95% CI, -0.97, 4.95) (Extended data, file 6).”*

*We have also noted this limitation in the Discussion section under ‘Strengths and limitations’:*

*“* *Limitations of our review were the inability to conduct a quantitative meta-analysis due to the substantial heterogeneity in study populations and methods including the tools used to assess neurobehavioural outcomes and definitions for iron status.* *Our secondary meta-analyses are likely to be limited by the small sample sizes and other unstandardized factors including age, dosage and duration of iron supplementation.”*

**Minor comment: Revise the first sentence under Methods "...metanalysis were" not "...metanalysis was"**

*Response: We have now edited the sentence under ‘Methods’ as follows:*

*“Our systematic review and meta-analyses were guided by the Preferred Reporting Items for Systematic Review and Meta-Analysis (PRISMA) guidelines* *and the protocol was registered on the PROSPERO database on 20 ^th^ March 2018 (registration number CRD42018091278).”*

# Peer review: Reviewer response for version 1

**DOI:** 10.21956/wellcomeopenres.18683.r44987

**Published:** 2021-08-06

**Authors:** Patricia Kitsao-Wekulo

**Affiliations:** 1 Maternal and Child Wellbeing Unit, African Population and Health Research Center, Nairobi, Kenya

The authors have presented data from a systematic review and meta-analysis which found mixed evidence for the effects of iron supplementation or fortification on neurobehavioural outcomes in children. This is an area on which there is limited evidence from Africa, despite the high prevalence of both iron deficiency and developmental delay. The authors are commended for the use of a comprehensive search strategy for their review which yielded important evidence on the effects of iron supplementation on neurobehavioural outcomes in African children, while at the same time comparing their findings with those from children living in non-African countries. 

 I have made the following comments for consideration by the authors:

**Introduction**

- The rationale for, and the objectives of the systematic review are clearly stated. However, I would like to suggest that the authors consider shifting some of the material in the Introduction. For instance, the second paragraph could be shifted to the beginning of this section as it gets straight to the point in providing context for the review.
- The first mention of 'randomised controlled trials' appears at the end of the introduction, and it is here that the abbreviation 'RCT' should appear after it has been written out in full. In later sections, the authors can just use the abbreviation, without having to spell it out in full again as they have done under the Methods and other sections.

**Methods**

- Under the subsection on the **search strategy,** the authors indicate that they 'searched...for studies published up to 9 ^th^ March 2021.' It is not clear which month/ year was considered as the start point.
- 'Disagreements between reviewers were resolved through discussion.' Please clarify what the disagreements were on and the content of the discussion to get to a consensus. 
- 'We used the revised Cochrane risk-of-bias tool for randomised trials...' This statement appears twice in the same paragraph. Consider revising.
- 'The large degree of diversity in the study variables necessitated narrative synthesis of the study findings.' Was this applied to all the studies included in the review, or only to those from Africa? Please clarify 

**Results**

- '...that were not relevant to our study.' I suggest you use 'review' rather than 'study'
- The information on the time period of the publications included in the review should be provided earlier, under search strategy
- 'Of the 34 RCT studies: 25 evaluated the effect of iron supplementation compared to placebo or no treatment; five the effect of iron-fortified foods compared to non-fortified foods; one the effect of fortification of formula milk with high compared to low dosages of iron; one the effect of immediate iron supplementation given concurrently or 28 days after antimalarial treatment on development in children with severe malaria and one the effect of varying and consistent doses of iron supplementation compared to placebo; two the effects of maternal iron supplementation on neurobehavioural outcomes in children after birth.'
  For clarity and to avoid repetition, the number of studies under each study description could be presented as Ns e.g. 'Among the 34 RCT studies were those that studied the effect of: iron supplementation compared to placebo or no treatment (N = 25); iron-fortified foods compared to non-fortified foods (N = 5); etc  
- Check consistency in the use of Sub-Saharan Africa or sub-Saharan Africa
- "Due to substantial heterogeneity in study methods, only seven (n=775) out of 24 studies that used the Bayley Scales of Infant Development (BSID) to assess cognitive development' - This statement is repeated under the subsection on **synthesis**.
- It is not clear what number of studies were included to investigate the effect of iron supplementation. Under the subsection on study characteristics, the number is indicated as 25. Later on, under 'cognitive development, the number is indicated as 23. Please clarify.
- Consider revising the following statement for improved readability as 'children' appears four times
  'In school children, one RCT of 119 Indonesian children reported improved IQ among children with IDA at baseline who received iron supplementation for three months compared to children who received placebo.'
- There is a lot of repetition in the text under the Results section which makes reading the text quite tedious. For instance, under the subsection on 'Educational achievement,' a similar statement appears in the third and sixth lines 'four studies (n=3058) reported no beneficial effects.' Consider revising
- The authors tend to use the word 'studies' several times in single statements and should consider revisions. For example, the first statement under the subsection titled 'Effect of duration..'
- The second objective for the review was ' **For comparison**, we further summarised evidence from randomised controlled trials on the effects of iron supplementation on neurobehavioral outcomes in non-African countries.' My sense is that the comparison does not really come out clearly in the results. The authors should consider providing a summary to provide this comparison, either at the end of each subsection on specific neurobehavioural outcomes or at the end of the Results section, if this fits within the journal guidelines.

**Discussion**

- In the first paragraph, there is discussion around the association between iron supplementation and the heightened risk of malaria infection. This information would fit better under the Introduction as it provides more context around why there are limited iron supplementation studies in Africa 

Are the rationale for, and objectives of, the Systematic Review clearly stated?

Yes

Is the statistical analysis and its interpretation appropriate?

I cannot comment. A qualified statistician is required.

Are sufficient details of the methods and analysis provided to allow replication by others?

Yes

Are the conclusions drawn adequately supported by the results presented in the review?

Yes

Reviewer Expertise:

Developmental psychology

I confirm that I have read this submission and believe that I have an appropriate level of expertise to confirm that it is of an acceptable scientific standard, however I have significant reservations, as outlined above.

# Response

**Published:** 2021-11-16

**Authors:** Agnes Mutua

**Affiliations:** KEMRI-Wellcome Trust Research Programme, Kenya

We thank the reviewer for her helpful comments, which we have addressed point-by-point below:

 The authors have presented data from a systematic review and meta-analysis which found mixed evidence for the effects of iron supplementation or fortification on neurobehavioural outcomes in children. This is an area on which there is limited evidence from Africa, despite the high prevalence of both iron deficiency and developmental delay. The authors are commended for the use of a comprehensive search strategy for their review which yielded important evidence on the effects of iron supplementation on neurobehavioural outcomes in African children, while at the same time comparing their findings with those from children living in non-African countries. 

 I have made the following comments for consideration by the authors:

**Introduction**

**The rationale for, and the objectives of the systematic review are clearly stated. However, I would like to suggest that the authors consider shifting some of the material in the Introduction. For instance, the second paragraph could be shifted to the beginning of this section as it gets straight to the point in providing context for the review.**

*Response: We agree with the reviewers point that the second paragraph provides a clearer context for our review and have now moved paragraph two under introduction to the beginning of the section as follows:*

*“Iron deficiency is the most common micronutrient deficiency affecting about two billion individuals globally and accounting for over 40% of all cases of anaemia ^1-3^* *. Children living in Africa disproportionately bear the highest burden of anaemia and iron deficiency. Approximately 43% of pre-school children are anaemic globally and over 60% of these children live in Africa ^1, 4^* *, while it is estimated that 52% of African children are iron deficient ^5^.*

**The first mention of 'randomised controlled trials' appears at the end of the introduction, and it is here that the abbreviation 'RCT' should appear after it has been written out in full. In later sections, the authors can just use the abbreviation, without having to spell it out in full again as they have done under the Methods and other sections.**

*Response: We have now abbreviated ‘randomised controlled trials’ in the last paragraph of the introduction and added the abbreviation RCT in the ‘Search strategy and eligibility criteria’, ‘Study selection’ and ‘Conclusions’ sections.*

**Methods**

**Under the subsection on the search strategy, the authors indicate that they 'searched...for studies published up to 9 ^th^ March 2021.' It is not clear which month/ year was considered as the start point.**

*Response: We thank the reviewer for this comment. For comprehensiveness, we did not restrict our search by date of publication, therefore we did not have a start date for the included studies. The earliest study we included was published in 1978. For clarification, we have now added the following statement in the ‘* *Search strategy and eligibility criteria’ section:*

*“The search was not restricted by language or publication date* *.”*

*Additionally, we repeated our search on 22 ^nd^ October 2021 and found one more RCT in Indian children. We have now updated the Abstract, Results section, Figures 1 and 2, References and Supplementary materials to reflect this addition.*

**'Disagreements between reviewers were resolved through discussion.' Please clarify what the disagreements were on and the content of the discussion to get to a consensus.**

*Response: The few disagreements were on the methods in some of the older studies. For clarification, we have now revised the sentence under the sub-section ‘Study selection, data extraction and quality appraisal’ as follows:*

*“Disagreements between reviewers on study methodologies were resolved through discussion following the pre-determined inclusion and exclusion criteria.”*

**

**'We used the revised Cochrane risk-of-bias tool for randomised trials...' This statement appears twice in the same paragraph. Consider revising.**

*Response: Thank you. The Cochrane risk of bias tool for randomised trials is only used to assess risk of bias in individually-randomised, parallel-group trials while the Cochrane risk of bias tool for randomised trials with additional considerations for cluster-randomised trials is an extended version that is only used to assess risk of bias in cluster RCTs. We have now revised the paragraph as follows:*

*“We used the revised Cochrane risk-of-bias tool for randomised trials (RoB 2) to assess for risk of bias for the individually-randomised, parallel-group RCTs included in the review. RoB 2 assesses five domains of bias including bias from the randomisation process, deviations from intended interventions, missing outcome data, and bias in measurement of the outcome and selection of the reported result. To assess the risk of bias in two cluster RCTs, we used the revised Cochrane risk of bias tool for randomised trials with additional considerations for cluster-randomised trials.”*

**'The large degree of diversity in the study variables necessitated narrative synthesis of the study findings.' Was this applied to all the studies included in the review, or only to those from Africa? Please clarify**

*Response: We summarized all the studies narratively and performed a secondary meta-analysis for seven studies in non-African countries that used the same tool (the Bayley Scales of Infant Development). We have now clarified that the meta-analysis was a secondary analysis and revised the sentence in the Methods section under ‘Synthesis of included studies’ as follows:*

*“The large degree of diversity in the study variables necessitated narrative synthesis of the study findings for all included studies.”*

*We have also revised the Methods section under ‘Secondary meta-* *analysis of seven studies in non-African countries’*

*“Due to the substantial variation in study methods, we did a secondary meta-analysis in a limited number of RCTs that all used the Bayley Scales of Infant Development (BSID) to assess cognitive and motor development in children living in non-African countries.* *None of the studies in African children used the BSID.* *”*

**Results**

**'...that were not relevant to our study.' I suggest you use 'review' rather than 'study'**

*Response: We have revised ‘study’ to ‘review’.*

**The information on the time period of the publications included in the review should be provided earlier, under search strategy**

*Response: Thank you, per comment 3 above, we have now clarified in the ‘Search strategy and eligibility criteria’ section’ that we did not restrict our search by date of publication and therefore we did not have a pre-determined start date for the included studies. Hence, we reported the time period of the included studies in the Results section.*

**'Of the 34 RCT studies: 25 evaluated the effect of iron supplementation compared to placebo or no treatment; five the effect of iron-fortified foods compared to non-fortified foods; one the effect of fortification of formula milk with high compared to low dosages of iron; one the effect of immediate iron supplementation given concurrently or 28 days after antimalarial treatment on development in children with severe malaria and one the effect of varying and consistent doses of iron supplementation compared to placebo; two the effects of maternal iron supplementation on neurobehavioural outcomes in children after birth.'**

**For clarity and to avoid repetition, the number of studies under each study description could be presented as Ns e.g. 'Among the 34 RCT studies were those that studied the effect of: iron supplementation compared to placebo or no treatment (N = 25); iron-fortified foods compared to non-fortified foods (N = 5); etc**

*Response: We have now edited the statement under ‘Study characteristics and outcomes’ in the Results as follows:*

*“Among the 35 RCTs were those that studied the effect of: iron supplementation compared to placebo or no treatment (n= 25), iron-fortified foods compared to non-fortified foods (n=5), formula milk fortified with high compared to low dosages of iron (n=1), immediate iron supplementation given concurrently or 28 days after antimalarial treatment on development in children with severe malaria (n=1), varying and consistent doses of iron supplementation compared to placebo (n=1), and maternal iron supplementation on neurobehavioural outcomes in children after birth (n=2).”*

**Check consistency in the use of Sub-Saharan Africa or sub-Saharan Africa**

*Response: Thank you. We have now revised ‘Sub-Saharan Africa’ to ‘sub-Saharan Africa’ in the sub-section ‘Comparison of studies in African countries versus non-African countries, and in the Discussion section for consistency.*

**"Due to substantial heterogeneity in study methods, only seven (n=775) out of 24 studies that used the Bayley Scales of Infant Development (BSID) to assess cognitive development' - This statement is repeated under the subsection on synthesis.**

*Response: We have rephrased the sentence in results under the ‘Cognitive development’ sub-section as follows:*

*“Secondary meta-analysis of seven studies (n=775) provided limited evidence of beneficial effects of iron supplementation on cognitive development in pre-school children (MD=1.73, 95% CI, -1.05, 4.52) (Extended data, file 5 ^21^ ).”*

**It is not clear what number of studies were included to investigate the effect of iron supplementation. Under the subsection on study characteristics, the number is indicated as 25. Later on, under 'cognitive development, the number is indicated as 23. Please clarify.**

*Response: We thank the reviewer for this comment. Two of the 25 studies did not investigate the effects of iron supplementation on cognitive outcomes and were therefore not included under the section on ‘Cognitive development’.*

**Consider revising the following statement for improved readability as 'children' appears four times**

**'In school children, one RCT of 119 Indonesian children reported improved IQ among children with IDA at baseline who received iron supplementation for three months compared to children who received placebo.'**

*Response: We have revised the sentence in the ‘Cognitive development’ section in results as follows:*

*“One RCT (n=119) in Indonesia reported improved IQ among school children with IDA who received iron supplementation for three months compared to those who received placebo ^54^.”*

**

**There is a lot of repetition in the text under the Results section which makes reading the text quite tedious. For instance, under the subsection on 'Educational achievement,' a similar statement appears in the third and sixth lines 'four studies (n=3058) reported no beneficial effects.' Consider revising**

*Response: Thank you. We have now removed the repeated text under ‘Educational achievement’ and throughout the Results section more generally and have made the Results more concise to improve readability.*

**The authors tend to use the word 'studies' several times in single statements and should consider revisions. For example, the first statement under the subsection titled 'Effect of duration..'**

*Response: We agree with the reviewer that the term ‘studies’ has been repeated in the Results section and we have removed the repeated text from the Results section. For instance, we have revised the sub-section ‘Effect of duration of supplementation or fortification’ in the Results as follows:*

*“In total, seven studies (n=534) investigated the effect of iron supplementation for less than three months and of these studies, five (n=419) reported beneficial effects of iron supplementation on neurobehavioural outcomes, while two (n=115) reported no beneficial effects. Out of 26 studies (n=8972) that evaluated the effect of iron supplementation (n=20) or fortification (n=6) for three months or more, 13 (n=3939) reported beneficial effects of iron supplementation (n=10) or fortification (n=3) on neurobehavioural outcomes and 13 (n=5033) reported no beneficial effects of iron supplementation (n=10) or fortification (n=3).”*

**The second objective for the review was 'For comparison, we further summarised evidence from randomised controlled trials on the effects of iron supplementation on neurobehavioral outcomes in non-African countries.' My sense is that the comparison does not really come out clearly in the results. The authors should consider providing a summary to provide this comparison, either at the end of each subsection on specific neurobehavioural outcomes or at the end of the Results section, if this fits within the journal guidelines.**

*Response: Thank you. We included a paragraph titled ‘Studies in African countries versus non-African countries’ in the Results after ‘Study characteristics and outcomes.’ We have now edited and moved this paragraph to the end of the Results and changed the title as follows for clarification:*

*“Comparison of studies in African countries versus non-African countries”*

*Five RCTs including a total of 1294 children evaluated the effect of iron supplementation or fortification on cognitive (n=4), motor (n=1), behavioural (n=1) or language (n=1) outcomes in African children. Only three out of the five studies were in sub-Saharan Africa and of the three one did not have a placebo group. Out of the five studies, two (n=647) reported beneficial effects on cognitive, motor or language outcomes only in anaemic children ^26, 27 ^while three (n=647) reported no beneficial effects in children ^24, 25, 28^. Of the three studies that reported no beneficial effect of iron supplementation, one included only children with ID ^24^, one had a prevalence of 21% of anaemia ^25 ^while one did not report baseline iron status* ^*25*^ *. Among children living in non-African countries, 30 RCTs including a total of 8694 participants investigated the effect of iron supplementation or fortification on neurobehavioural outcomes. Compared to studies in African children, over half of the 30 studies reported beneficial effects on neurobehavioural outcomes, mostly in children with ID, IDA or anaemia ^29–57^.”*

*Additionally, under each neurobehavioural outcome, we discuss African studies first, then studies from non-African countries. We have now added the following information in the methods section under the subsection ‘Synthesis of included studies’:*

 “ *Under each neurobehavioural domain, we first summarized studies in African countries followed by studies in non-African countries.”*

**Discussion**

**In the first paragraph, there is discussion around the association between iron supplementation and the heightened risk of malaria infection. This information would fit better under the Introduction as it provides more context around why there are limited iron supplementation studies in Africa**

*Response: Thank you for this comment. We have now added this information to the last paragraph of the introduction section as follows:*

*“* *Despite the high prevalence of both iron deficiency and anaemia, there are few studies investigating the effects of giving iron on neurobehavioural outcomes in African children. This may be due to many factors including concern that iron supplementation may increase the risk of malaria and other infections ^18-20^.”*
